# Consumption: Climatic Treatment
**The Climatic Treatment of Consumption*. By J. A. Lindsay, M.D. London: Macmillan and Co.


**Published:** 1887-10-01

**Authors:** 


### Reviews and Notices.

### CONSUMPTION: CLIMATIC TREATMENT.*

Consumption and its management give occasion for the profoundest study and the most conscientious professional conduct. In no department of medical activity is it easier to play the charlatan and the greedy knave. So numerous are the cases of phthisis, and so widely sweeping is the deadly range of the disease, that its prominent features and fatal characteristics are universally known. No plague-spot, visible in the face of a stricken man, gives more hopeless assurance of impending doom than the well-marked appearances of pulmonary consumption. The beholder, looking at his fellow-mortal, involuntarily and almost unconsciously assigns him to an early grave. That being so, the best feelings of humanity are roused on behalf of the sufferer; and those whose business it is to deal with the physical ills of mankind bring all their energies to bear upon the difficult work of removing, or at least alleviating, the serious and portentous symptoms of the disease. That man is a monster who can attend a consumptive patient without being moved in the profoundest depths of his being to use every possible effort for saving the life of his sinking brother-man. It is an agonising moment when a fellow-being who cannot swim is cast into the engulfing waves with no help at hand. But the agony is only for a moment. With the consumptive the death-struggle may last for years, whilst fears and hopes alternately possess the mind.

It need occasion no surprise, therefore, that a considerable number of medical men should devote their lives to the study of this one subject; and it is equally easy to understand why some of them, finding the prospects of success in the management of it very doubtful, should learn to think it justifiable to deceive their patients with flattering hopes rather than, by simplicity and truthfulness, to prepare their minds for the too often inevitable end. We do not affirm that every man with phthisical symptoms will certainly die of consumption. Far from it! Many persons who have undoubtedly been the subjects of advanced lung-disease of a consumptive character have permanently recovered, and many more will do so in the future. But whilst this is freely admitted, it nevertheless remains painfully true that the majority of such cases die, and that in spite of the highest individual or combined medical skill which the world affords. He who teaches otherwise, and professes to cure all or a large proportion of consumptive persons, is either hopelessly ignorant or a wilful deceiver, and deserves punishment at the hands of the law rather than confidence and prosperity.

There is no specific, or " cut and dried" cure for consumption known to science; still less is there any such cure known outside science. This is true, whatever view be taken of the etiology and pathology of the disease. The germ pathologists know no better methods of cure than their immediate predecessors. The believers in special remedies are no more successful in practice than those who treat on general principles. In the experience of both the victories of treatment are few, the defeats overwhelmingly numerous. An almost infinite variety of methods have been tried in practice at different times. One man believes in internal treatment, another in external; one in medical and another in surgical. Now a warm climate is recommended, and then a cold one. One doctor advises this place, another that; one prefers sea-air, and his neighbour the air of the mountains. Every book which is written on the subject differs in some more or less important particulars from every other. One general affirmation may, however, be made concerning them all, viz., that they have each contributed something towards the elucidation of the subject, and that nearly every one of them has words to say about the treatment of the disease which it is important for the practitioner to know. He probably will be most skilful at the bedside who has few fixed methods, but takes every case on its own merits, and brings to bear upon it all the knowledge which books have taught him, together with the choicest stores of his own accumulated experience. He is the true man of science, and if any given case be in itself curable, and be placed under his care at a sufficiently early stage, it may be taken for granted that that particular patient is in the most favourable possible circumstances for recovery.

The book before us does not profess to be an exhaustive treatise on consumption and its treatment. It deals with one department of practice, and brings together a vast amount of reliable information. Among all the factors that go to make up a *modus operandi* of cure, climate is perhaps the chief. Drugs, which are the sheet-anchor of the charlatan, sink into insignificance in comparison with it. Almost every climate has in turn been tried, and had its advocates and admirers. The humid, the dry, the hot, the cold, the sea, the mountains, the simple, and the mixed. The influence of rainfall, of vegetation, of altitude, of wind currents—all have been studied with earnestness and intelligence. The results of much labour on the part of others, and of much travel and many personal observations on Dr. Lindsay's own part, are here rendered available for the use of those practitioners who cannot make the tour of the world for themselves. As will be anticipated by readers familiar with recent opinion on the subject, Dr. Lindsay gives the preference to mountain climates over every other. A high altitude, with a dry soil, and vegetation such as is usually

* *The Climatic Treatment of Consumption.* By J. A. Lindsay, M.D. London: Macmillan and Co.

found in such regions, he considers to possess atmospheric conditions more favourable to the consumptive than any other circumstances. He advocates a life in the open air, with vigorous exercise and as robust and nourishing a diet as can by any possibility be assimilated. All the principal sanatoria of both hemispheres are brought into review, and the special characteristics of each pointed out and adjudicated upon. It cannot be claimed that the information is always the fullest which might have been obtained, or that the judgments are such as it is impossible to call in question. But it may be said of the book that it displays great industry, and a conscientious desire to be impartial, with an equally sincere and not unsuccessful effort to be of service to the reader. Whoever wishes to bring his knowledge of climates and sanatoria down to date will be well-advised to make himself the possessor of this short, readable, and inexpensive volume.

